# Nanoengineered injectable hydrogels derived from layered double hydroxides and alginate for sustained release of protein therapeutics in tissue engineering applications

**DOI:** 10.1186/s12951-023-02160-2

**Published:** 2023-11-02

**Authors:** V. H. Giang Phan, Hai-Sang Duong, Quynh-Giao Thi Le, Gopinathan Janarthanan, Sanjairaj Vijayavenkataraman, Hoang-Nam Huynh Nguyen, Bich-Phuong Thi Nguyen, Panchanathan Manivasagan, Eue-Soon Jang, Yi Li, Thavasyappan Thambi

**Affiliations:** 1 Biomaterials and Nanotechnology Research Group, Faculty of Applied Sciences, Ton Duc Thang University, Ho Chi Minh City, Vietnam; 2 The Vijay Lab, Division of Engineering, New York University Abu Dhabi, Abu Dhabi, United Arab Emirates; 3 Department of Mechanical & Aerospace Engineering, Tandon School of Engineering, New York University, Brooklyn, NY 11201 USA; 4 Department of Applied Chemistry, Kumoh National Institute of Technology, Daehak-ro 61, Gumi, Gyeongbuk 39177 Republic of Korea; 5 College of Materials and Textile Engineering & Nanotechnology Research Institute, Jiaxing University, Jiaxing, 314001 Zhejiang People’s Republic of China; 6 Graduate School of Biotechnology, College of Life Sciences, Kyung Hee University, Yongin si, Gyeonggi do 17104 Republic of Korea

**Keywords:** Injectable hydrogels, Erythropoietin, Sustained release, 3D printing, Tissue engineering

## Abstract

Chronic Kidney Disease (CKD) which involves gradual loss of kidney function is characterized by low levels of a glycoprotein called Erythropoietin (EPO) that leads to red blood cell  deficiency and anemia. Recombinant human EPO (rhEPO) injections that are administered intravenously or subcutaneously is the current gold standard for treating CKD. The rhEPO injections have very short half-lives and thus demands frequent administration with a risk of high endogenous EPO levels leading to severe side effects that could prove fatal. To this effect, this work provides a novel approach of using lamellar inorganic solids with a brucite-like structure for controlling the release of protein therapeutics such as rhEPO in injectable hydrogels. The nanoengineered injectable system was formulated by incorporating two-dimensional layered double hydroxide (LDH) clay materials with a high surface area into alginate hydrogels for sustained delivery. The inclusion of LDH in the hydrogel network not only improved the mechanical properties of the hydrogels (5–30 times that of alginate hydrogel) but also exhibited a high binding affinity to proteins without altering their bioactivity and conformation. Furthermore, the nanoengineered injectable hydrogels (INHs) demonstrated quick gelation, injectability, and excellent adhesion properties on human skin. The in vitro release test of EPO from conventional alginate hydrogels (Alg-Gel) showed 86% EPO release within 108 h while INHs showed greater control over the initial burst and released only 24% of EPO in the same incubation time. INH-based ink was successfully used for 3D printing, resulting in scaffolds with good shape fidelity and stability in cell culture media. Controlled release of EPO from INHs facilitated superior angiogenic potential *in ovo* (chick chorioallantoic membrane) compared to Alg-Gel. When subcutaneously implanted in albino mice, the INHs formed a stable gel in vivo without inducing any adverse effects. The results suggest that the proposed INHs in this study can be utilized as a minimally invasive injectable platform or as 3D printed patches for the delivery of protein therapeutics to facilitate tissue regeneration.

**Supplementary Information:**

The online version contains supplementary material available at 10.1186/s12951-023-02160-2.

## Introduction

**Scheme 1:**
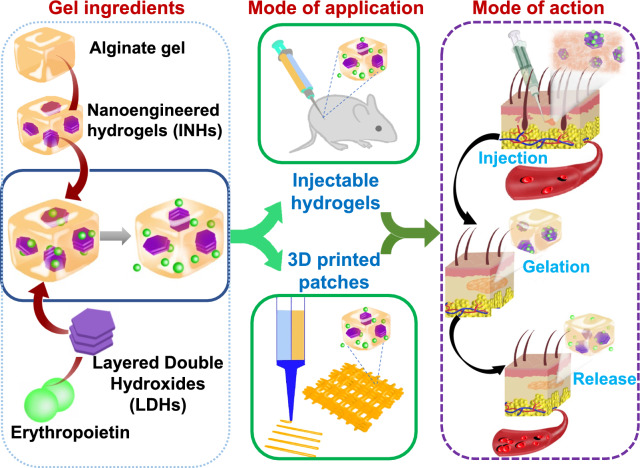
Working principle of INHs. Preparation of the EPO-loaded INHs from alginate gel and LDHs and their utilization in injectable hydrogel and 3D printing applications. Effect of sustained release on tissue regeneration after in situ implantation of INHs at the subcutaneous tissue of mice

Hydrogels are three-dimensional hydrated networks that are generally used in tissue engineering for their beneficial properties such as biocompatibility, biodegradation, interconnected pores, waste removal, nutrient transport, etc. [1–3]. Due to high biocompatibility and adjustable porous network structures, hydrogel materials get great consideration for drug entrapment applications [4–6]. Among the reported biomaterials, alginate is an ideal candidate and it has been successfully used as a three-dimensional matrix for protein/cell encapsulation [7–9]. Sodium alginate, a polysaccharide found in brown seaweed composed of β-D-mannuronic acid (M) and α-L-guluronic acid (G), is famous for gel-forming properties with divalent cations connecting the G-blocks to form the “egg-box” structure [10, 11]. Despite its desirable properties such as biocompatibility, low toxicity, and adsorption capacity, alginate has a drawback related to the uncontrolled degradation of the gel when exposed to calcium chelating agents [12, 13]. This limitation hinders the precise control release of drugs, leading to increased side effects and the need for frequent administration when used for drug delivery applications (Scheme 1).

To control the degradation and enhance mechanical properties of such injectable hydrogels, a wide range of nanostructured materials, such as inorganic, polymeric, carbon-based, and metallic nanomaterials, have been integrated into the mesh of the hydrogel network [14–21]. Particularly, the incorporation of two-dimensional (2D) nanomaterials with significantly high surface area in comparison to conventional polymeric nanoparticles has shown exceptional improvement in mechanical strength because of their ultrathin 2D structure [22–24]. 2D nanomaterials, characterized by their high structural anisotropy and surface-to-volume ratio, can effectively interact with various polymers, leading to the formation of stiff and tough hydrogels [25–27]. While various 2D nanomaterials have been employed to investigate the controlled release and mechanical properties of hydrogels [28], the utilization of lamellar inorganic solids with a brucite-like structure for controlling the release of protein therapeutics has not been previously reported. Lamellar inorganic solids have been widely utilized in numerous medical applications, including controlled drug delivery and tissue engineering [29, 30].

Generally, to control the drug release and improve mechanical properties of SA and Ca^2+^ gel, layered double hydroxides (LDH) were introduced to the alginate hydrogel (Alg-Gel) network to obtain nanoengineered injectable hydrogels (INHs) [31]. As one type of lamellar materials that can be written as Mg_6_Al_2_(OH)_16_](CO_3_)0.4(H_2_O) [31, 32], LDHs contain layers that are positively charged due to bivalent (Mg^2+^ for example) and trivalent (Al^3+^) ions. Through the intercalation of anions, the layered structure is neutralized [33]. The unique structure allows biological agents that are negatively charged to form nanohybrids by exchanging ions [34]. This LDH was synthesized using a simple co-precipitation method. To showcase the release properties of the newly developed nanoengineered injectable hydrogel system, we have chosen a model glycoprotein called Erythropoietin (EPO). This glycoprotein hormone consists of 165 amino acid residues which are specifically secreted by the peritubular cells of the kidneys [35–37]. The EPO hormone functions as a chemical signal that stimulates the bone marrow to produce red blood cells (RBCs) [38]. The average life span of RBC is approximately 115 days [39]. In order to balance levels of RBCs for a normal life function, the bone marrow generates 2.5 million reticulocytes every second [40]. Chronic kidney disease (CKD) involves a gradual decrease of kidney function, which led to low EPO levels as well as RBC deficiency and development of anemia [41–43]. Since the 1980s, patients with CKD have generally been treated with recombinant human erythropoietin (rhEPO), which is administered exclusively through subcutaneous injections. The utilization of rhEPO not only significantly elevates hemoglobin levels but also reduces the requirement for frequent blood transfusions [44].

Typically, rhEPO injection solution available on the market has short half-lives (4 h for intravenous and 8 h for subcutaneous injection), and demands frequent administration of 1 to 3 times weekly [45, 46]. Additionally, high endogenous EPO levels could increase risks of side effects such as thromboembolic events [47], cardiovascular complications [48], seizures [49], pulmonary embolism [50], and mortality [51]. Therefore, attempts have been paid to control and sustain the release of EPO so as to lessen the adverse effects and reduce frequency of injection, resulting in improved patients’ compliance [52–54].

Taking these factors into consideration, we have designed the injectable nanoengineered hydrogels (INHs) with the purpose of incorporating EPO and effectively controlling its release (Scheme 1). Scanning electron microscopy (SEM) was used to observe effective dispersion of LDH nanoparticles within the Alg-Gel. Biocompatibility and angiogenic potential of hydrogel were examined systematically by various tests including MTT (3-(4, 5-dimethylthiazolyl-2)-2, 5-diphenyltetrazolium bromide) assay, hemolysis, and chick embryo chorioallantoic membrane (CAM) assay. The improved mechanical property and stability of INHs was verified by 3D printing and post-printing stability was examined upon incubation in culture medium and buffer solutions. The loading and controlled release property of INHs was demonstrated using EPO. The EPO released from INHs was detected and quantified using Bradford protein assay. Finally, in situ gel formation was examined using mice models. The INHs exhibited enhanced mechanical properties and the mechanical stiffness of the biomaterials control the release of EPO in a sustained manner. Prolonged release of EPO could potentially reduce the frequent injections and improve patient compliance. Considering the tunable properties of the hydrogel, the INHs can be employed as drug delivery depot in various biomedical applications, including tissue engineering and regenerative medicine applications, which could offer significant advantages over conventional hydrogel formulations.

## Materials and methods

### Materials

SA, magnesium chloride hexahydrate (MgCl_2_.6H_2_O), aluminum chloride hexahydrate (AlCl_3_.6H_2_O), and rhEPO were obtained from Sigma-Aldrich (St. Louis, MO, USA). Phosphate buffered saline pH 7.4 (PBS) and anhydrous calcium chloride (CaCl_2_, 98%) were purchased from Merck (Darmstadt, Germany). All other chemicals were purchased from Sigma-Aldrich (St. Louis, MO, USA) and were used without any purification.

### LDH nanoparticle synthesis

Co-precipitation method was used to synthesize LDH nanoparticles [55, 56]. The reagents used were MgCl_2_.6H_2_O (6.82 g) and AlCl_3_.6H_2_O (4.04 g). The reagents were dissolved in 100 mL of deionized water. The reaction solution pH was adjusted to pH 9.0 by slowly adding 1 M NaOH. Then, the mixture was magnetically stirred at 80 °C for 48 h in a two-neck flask before being washed several times with distilled water by centrifugation (10,000 rpm, 15 min) until no Cl^−^ could be detected by Ag^+^ (this confirmation test was carried out to verify the complete removal of MgCl_2_ and AlCl_3_ salts from the LDH nanoparticles), and finally dried at 60 °C in a hot air oven to obtain white LDH powder.

### Investigation of gelation time

To establish a hydrogel network, it is crucial to study the optimal gelation time of sodium alginate (SA) when crosslinked with calcium ions (Ca^2+^) for subcutaneous injection [57]. The hydrogel prepared using only SA with Ca^2+^ was denoted as Alg-Gel. In brief, SA solution with the final concentration of 4% (w/v) was prepared by stirring appropriate quantity of SA powder in deionized water and transferring into different tubes. Furthermore, each tube of SA solution was mixed with Ca^2+^ solution with different concentrations (0.005, 0.01, 0.015, 0.02, 0.025, 0.03 mol/L) at a volume ratio of 2:1 to obtain Alg-Gel. Additionally, the gelation time was measured using a stopwatch to identify the combination that achieves an optimal gelation time for intravenous administration. For the preparation of INHs, the SA solution 4% (w/v) was stirred with LDHs (0.125, 0.25, 0.5 mg/mL) for 1 h and placed in an ultrasonic bath for another 1 h. Subsequently, the combinations were mixed with the ideal Ca^2+^ concentration found in the previous experiment (i.e., 0.02 mol/L). The LDHs/SA/Ca^2+^ hydrogels (denoted as INHs) were at the volume ratio of 1:4:2 and all the concentrations measured were final concentrations. Based on the amount of LDHs in the hydrogels, the samples were grouped as LDHs (0.125 mg)/SA/Ca^2+^ (denoted as INHs-0.125), LDHs (0.25 mg)/SA/Ca^2+^ (denoted as INHs-0.25), and LDHs (0.5 mg)/SA/Ca^2+^ (denoted as INHs-0.5). The hydrogel prepared without LDHs was labeled as Alg-Gel and served as the control in the study.

### Characterization methods

#### Fourier transform infrared (FTIR) spectra

FT-IR spectrophotometer (Spectrum Two FT-IR Spectrometer, PerkinElmer, UK) was used to detect the formation of LDHs, LDHs/EPO complex, and INHs with different concentrations of EPO. The IR spectrum for all samples was recorded within the range of 4000–500 cm^−1^.

#### Scanning electron microscope (SEM) imaging

The surface morphological characteristics of Alg-Gel and INHs were evaluated using SEM (Hitachi S-4800) imaging. The samples were frozen and freeze dried using a lyophilizer (Martin Christ, Alpha 1–2 LD plus) before the gold sputter coating.

#### Transmission electron microscopy (TEM)

The morphology and size distribution of LDHs nanoparticles suspension were observed by utilizing a TEM system (TEM, JEM 1400, JEOL, Japan) operated at 200 kV accelerating voltage.

#### X-ray diffraction (XRD)

XRD (Rigaku D/Max-2400 XRD) was used to analyze the molecular structure of LDHs and EPO-LDHs. For XRD analysis, the freeze-dried gels were placed between cover glasses and subsequently transferred to the sample holder. The samples were scanned in the range of diffraction angle 2θ = 5–80° with a step size of 0.02 and a scan speed of 0.4 s. The X-ray radiation was generated from Cu-Ka source with a wavelength (λ) of 0.154 nm.

#### Zeta potential

The electrical potential (ζ) was measured using Zetasizer Nano ZS (Malvern Instrument, UK). For zeta potential measurement, the samples were prepared in deionized water and stirred at room temperature for 1 h before being subjected to analysis.

#### Thermogravimetric analysis (TGA)

Thermal stability of Alg-Gel and INHs was recorded using a TA SDT 2960 (TA Instruments Co., New Castle, DE 19720, United States). In order to measure the TGA curve, samples were placed in alumina pans and heated between 25 °C and 700 °C temperature range under nitrogen atmosphere (flow rate: 100 mL/min) with a heating rate of 10 °C/min. All the samples were frozen and freeze dried as mentioned previously.

### Mechanical testing

The stress–strain tests were carried out to measure the compressive strength of hydrogels by using a Universal Testing Machine (Instron 3343, Boston, MA, USA) equipped with a 1 kN load cell at a crosshead speed of 10 mm/min. To examine the mechanical improvement of hydrogels, two different INHs were prepared with different amounts of LDHs, INHs-0.125 and INHs-0.25 respectively. Cylindrical hydrogel constructs with a diameter of 12 mm and height of 15 mm were positioned between self-leveling plates and subjected to compression at a rate of 10 mm/min.

### Swelling test

The swelling ratio of hydrogels was examined using gravimetric method. For this study, the dried hydrogels (*W*_o_) were immersed in phosphate-buffered saline (PBS, pH 7.4) at 37 °C. At specific time points, the swollen samples were taken out and weighed (*W*_t_) after removing the excessive PBS with filter paper. The percentage of swelling degree was calculated using the following formula:

$$Swelling \, ratio \left(\%\right)=\frac{{{W}_{t}- W}_{o}}{{W}_{o}}\times 100$$

### 3D printing of different patch (lattice) structures

Square shaped 3D structures were loaded in STL form and the dimensions as well as infill percentages were adjusted to obtain lattice patterns with varying strut numbers (15 mm × 15 mm and 30 mm × 30 mm) on Regemat 3D designer software and sliced to obtain the 3D printable model. The 3D printable model was viewed and the object was moved to the desired position and sliced again when required. The generated G-codes were cross checked in the advanced option of the software and subsequently printed using the connected Regemat Reg4life 3D bioprinter. After software configuration, the printing head (T0/T1) and the platform were autoconfigured and checked for extrusion using the advanced options. Printing parameters such as nozzle size, layer height, and flow rate, were modified to get optimized flow of hydrogel from the nozzle tip. After optimization, the process parameters chosen for all printed structures were a layer height of 300 μm and a flow speed of 2.30 mm/s with a 25G needle. For printing, the hydrogel inks were loaded into a 10 mL plastic syringe with a 25G plastic conical needle and printed onto a glass coverslip/petri plate. The printed structures were immersed in Dulbecco's Modified Eagle Medium (DMEM) cell culture medium/PBS (7.4 pH) or water to check its stability and shape fidelity. At regular intervals, the printed struts were observed for swelling and degradation or collapse of the structure. This 3D printing experiment was demonstrated to show the ability of the hydrogels to be printed and its potential to be used as 3D-printed personalized drug delivery systems.

### Loading and release of EPO in vitro

Two types of hydrogel formulation samples with and without LDHs were prepared to examine the influence of LDHs on controlled release of EPO. Specifically, the first group of samples contained 4% SA solution (wt/v), 0.25 mg/mL LDHs, 62.5 IU of EPO, and 0.02 mol/L of Ca^2+^ with a volume ratio of 4:1:1:2 (EPO-INHs-0.25). The second group included the same components and concentrations as the first ones, but 0.25 mg/mL LDHs were substituted with deionized water (EPO-Alg-Gel). 2 mL of PBS solution was added to the hydrogels before they were placed at 37 °C water bath with 20 rpm; 1 ml of samples was withdrawn at specific time points (after 2, 4, 6, 12, 18, 24, 36, 48, 60, 72, 84 and 108 h). Each time, they were replaced with 1 mL of fresh PBS medium. The EPO concentrations in each sample were determined by the Bradford method, and this investigation had been repeated three times.

### In vitro biocompatibility

MTT colorimetric assay was conducted to evaluate the cell proliferation of Alg-Gel and INHs. The samples placed in 24 well plates were exposed to UV irradiation for 20 min for sterilization. HaCaT keratinocytes and BLO-11 mouse muscle fibroblast cells were seeded on them at a density of 50 × 10^3^ cells/well. After incubation for 48 h, 50 µl of MTT dye was added to the wells. The supernatant was transferred to 96 well plates after 4 h of incubation and the absorbance was read at 570 nm by using BioTek Epoch 2 microplate reader.

### Live-dead cell imaging

Viability of BLO-11 mouse muscle fibroblast cells on Alg-Gel and INHs-0.25 (2000 µg/mL) were evaluated using live-dead staining assay. In 24 well plates, the cells were cultured in DMEM medium for 48 h and then stained with Live/Dead assay kit (Invitrogen R37601). The staining procedure was carried out as per the manufacturer’s protocol. A Nikon Confocal A1R microscope was used to obtain images.

### Chick embryo chorioallantoic membrane (CAM) assay

CAM assay was performed to evaluate the angiogenesis potential of three groups of hydrogels including EPO-Alg-Gel, EPO-Alg-Gel and EPO-INHs-0.25. A small hole was created on the 13th day of fertilized chicken eggs and the gel was implanted on the CAM surface. To ensure the healthy development of the embryo, the egg hole was covered with Parafilm and maintained in a sterile environment with a temperature of 37 °C and humidity of 70%. On day 15 and day 17, the implanted hydrogels were visually inspected and imaged to assess the extent of angiogenesis. The number of blood vessels and vascularized area around the hydrogels were quantified using ImageJ software.

### Hemolysis assay

The hemocompatibility of the hydrogels was determined using a hemolysis assay. The red blood cells (RBCs) were isolated by centrifuging the blood collected from mice at 3000 rpm for 20 min. The obtained RBCs were washed twice with PBS and diluted in PBS to obtain a final concentration of 10% (v/v). Subsequently, 500 µL of RBCs were added to 500 µL of hydrogel formulations [20% (wt/v)] and incubated at 37 °C for 3 h. Identical volume of RBCs incubated in PBS and Triton-X (0.1%) served as negative and positive controls respectively. The hydrogels exposed with RBCs were centrifuged at 3000 rpm for 10 min and supernatants (200 µL) were transferred to 96-well plates. Using a microplate reader, the absorbance of the supernatants was measured at 540 nm [58]. The hemolysis percentage was calculated with the following equation:

$$Hemolysis\left(\%\right)=\frac{{OD}_{sample}-{OD}_{negative \, control}}{{OD}_{positive \, control}-{OD}_{negative \,control}}\times 100\%$$

### In vivo hydrogel formation

Albino mice (16–20 g) were used to determine the in vivo injectability of the gel. Separate blends of LDHs (0.25 mg)/EPO/SA and Ca^2+^ solution (EPO-INHs-0.25) was prepared and transferred to a dual barrel syringe. The mixture was then subcutaneously administered into the dorsal region of the mice. After 30 min, the mice were sacrificed and the gels were recovered for further investigation. This animal experiment, conducted in accordance with the guidelines of Jiaxing University, received approval from the Institutional Committees of Jiaxing University.

### Statistical analysis

All experiments were done in triplicates and the experimental data were expressed as mean values with their corresponding standard deviations. Statistical analysis was conducted using one-way ANOVA, and the significance of the results was evaluated using Tukey's test (p < 0.05) with OriginPro 9.0 software (OriginLab. Corp., USA).

## Results

### Synthesis of LDHs

**Fig. 1:**
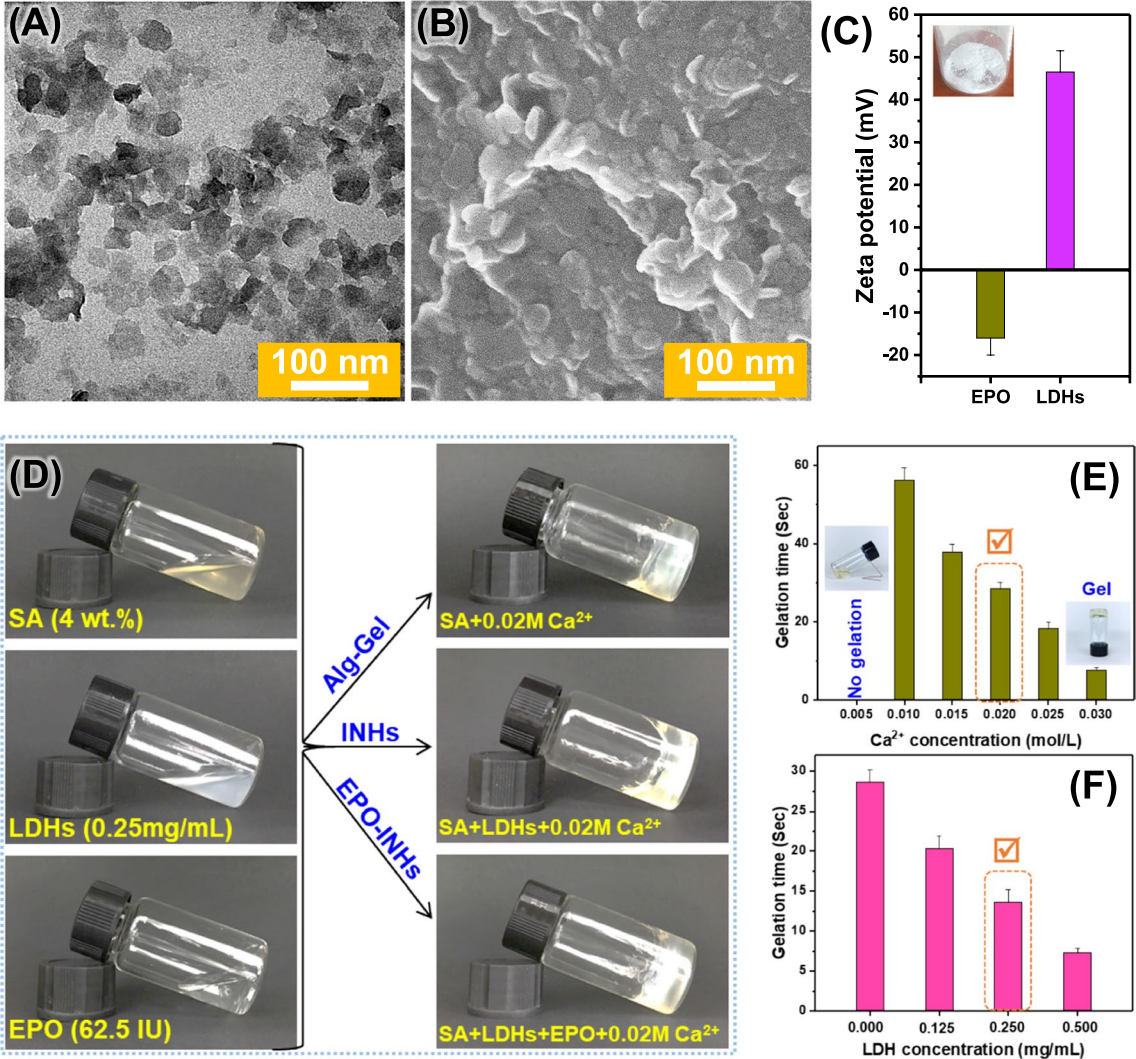
Characterization of LDHs. **A** TEM image of LDHs. **B** SEM image of LDHs. **C** Zeta potential of EPO and LDHs dispersed in deionized water. Inset photograph is for the dried LDHs. **D** Formation of hydrogels. Representative formation of SA-based hydrogels with LDHs and EPO in the presence of fixed Ca^2+^ concentration (0.02 mol/L). **E** Gelation time of Alg Gel with different Ca^2+^ concentrations. Inset photographs are for sol to gel phase transition of SA polymer with increasing Ca^2+^ concentrations. **F** Gelation time of Alg Gel with increasing concentration of LDHs to prepare INHs. Error bars in the graph represents mean ± SD (n = 3)

Based on the co-precipitation method, LDHs have been fully synthesized from AlCl_3_ and MgCl_2_ [59]. The dried LDH powder effectively dispersed in PBS from low to high concentrations (1 mg/mL to 5 mg/mL). More importantly, the dispersed LDH nanoparticles showed good colloidal dispersion, maintained for at least a week without aggregation. Nanoparticles refer to solid spheres of size ranging from 10 to 1000 nm. Often, nanoparticles are composed of biodegradable and biocompatible polymers of synthetic or natural origin [60], in which bioactive drugs are entrapped into the particle matrix during the synthesis process or post process [61]. In this study, TEM analysis was used to measure the LDH particle size and morphology. As shown in Fig. 1A, LDHs have sizes in the range of 40–80 nm, which is an ideal size for the controlled delivery application. Furthermore, SEM images clearly demonstrated the successful synthesis of plate-like LDHs (Fig. 1B). The zeta potential mean is + 46.5 mV, revealing that the LDHs can form a complex with EPO, which is a negatively charged glycoprotein (Fig. 1C).

### LDHs/Alg gelation time

Determining gelation time is important factor in injectable systems that transforms from a free-flowing solution to an immovable gel with shape fidelity [62]. Figure 1D–F show the gel photograph and gelation time of SA in the presence of increasing concentrations of Ca^2+^ ions. Particularly, the concentrations below 0.005 mol/L do not seem to form a hydrogel. As shown in Fig. 1E, the gelation times decrease gradually with the increase of Ca^2+^ concentrations. Clinically, in the in vivo practice, Ca^2+^ concentration of 0.02 mol/L is chosen as an ideal for gel formation in a living body [63]. Therefore, the gel prepared using SA solution [4% (wt./v)] with 0.02 M Ca^2+^ solution has been chosen for developing INHs.

For the optimization of INHs, different concentrations of LDHs (0.125, 0.25, and 0.50 mg/mL LDHs) were mixed with optimized concentrations of SA solution [4% (wt/v)] with 0.02 M Ca^2+^ solution and gelation time was recorded (Fig. 1F). Obviously, the association of LDHs in the formulation showed a shorter gelation time in comparison to LDH-free hydrogel. This is due to the electrostatic interaction between abundant carboxylate anions present in the SA and positively charged LDHs that increase the viscosity of the gel, thereby shortening the gelation time. During gelation, SA/LDHs complex form a structures in which two alginate layers envelope the LDHs [64]. In general, the alginate chains envelope the LDHs by the ionic interaction. At certain concentration, the formation of bilayer arrangement of the alginate molecules into a layered structure [65]. The combination of 0.25 mg/mL LDH final concentration and 4% SA with the 0.02 mol/L Ca^2+^ is chosen for the next experiment (i.e., INHs-0.25 in which the value 0.25 indicates the amount of LDHs in mg/mL).

### Injectability and shape moldability

**Fig. 2:**
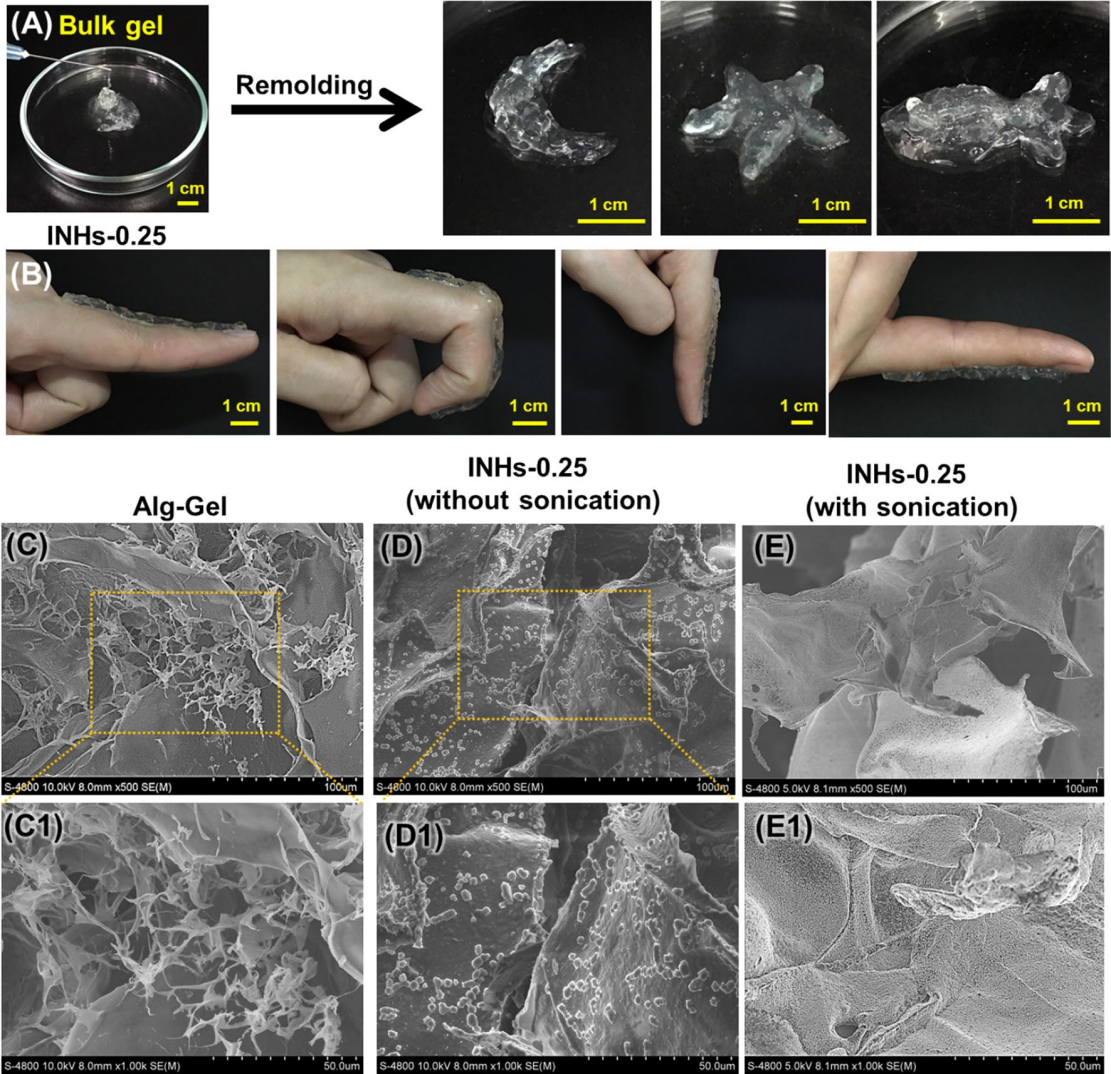
Injectability and moldability. **A** INHs can be extruded from a hypodermic needle (25G) and can form a bulk gel. The INHs can also be remolded into different shapes (e.g., Moon, star and fish). **B** The remolded hydrogel can adhere to the skin and adapt the finger’s movements. SEM image of **C** Alg Gel and (**D** and **E**) INHs with and without sonication. C1, D1 and E1 are the corresponding magnified images of **C**, **D** and **E** respectively. The INHs hydrogel prepared with sonication showed effective dispersion within the porous network of hydrogels

Injectable hydrogels, which are beneficial to carry therapeutic agents due to their injectability with minimal tissue damage, can fill irregular shapes when administrated in an intended site [66]. As shown in Fig. 2A, INHs-0.25 can be smoothly extruded in the form of a bulk gel, proving the potential for being used as injectable biomaterials. Thanks to their crosslinked structure, the hydrogels were tested to provide viscous sharp drops, which can be purposely remolded into any shape [67]. As shown in Fig. 2B, this biomaterial can adhere to human skin, and its viscous drop being extruded from a syringe can be easily shaped on the skin and adapt to any movements of the finger. Overall, the hydrogels appear to have injectability, re-moldability, and tissue adherence characteristics which are suitable for being used as an injectable carrier of protein therapeutics.

### Morphology of hydrogels

The porous properties of the hydrogels affect cell infiltration, proliferation, and its function. Therefore, SEM imaging of freeze-dried hydrogels was used to investigate the porous structure of the hydrogels. As shown in Fig. 2C, C1, the Alg-Gel showed porous structure with interconnected pores, mimicking the honeycomb-like structures. The bioengineered INHs-0.25 prepared by the introduction of LDHs, the interaction between LDHs and SA increased the physical cross-linking density, causing the pores to become slightly larger (Additional file 1: Fig. S1). The molecular entanglement between SA and LDHs was strengthened by the combination of ionic and hydrophobic interactions, leading to the formation of strong physically cross-linked hydrogel network. This could enhance the mechanical properties of the hydrogels. Particularly, the hydrogel prepared without sonication (Fig. 2D, D1) exhibited less effective dispersion of LDHs within the micropores, whereas the INHs-0.25 hydrogels prepared with sonication (Fig. 2E1, E2) showed more efficient dispersion of LDHs. The dispersion of LDHs within the hydrogel network was confirmed through elemental mapping of the SEM images (Additional file 1: Fig. S2). Elemental analysis demonstrated that uniform distribution of elemental Al and Mg in the hydrogel network.

### Characterization of EPO embedded LDHs (LDHs/EPO)

**Fig. 3:**
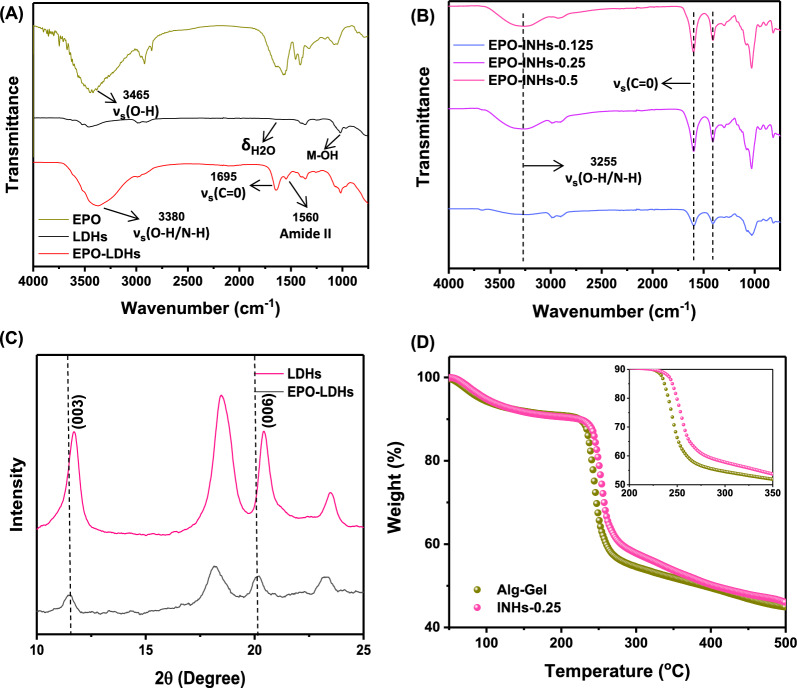
Characterization of EPO-loaded LDHs. **A** and **B** FT-IR spectra of LDHs, EPO-LDHs, and EPO-INHs. **C** XRD pattern of LDHs and EPO-LDHs. **D** TGA of Alg Gel and INHs

The FT-IR spectra of synthesized LDHs are shown in Fig. 3A. The strong band from 3600 to 3100 cm^−1^ is attributed to the stretching vibrations of the hydroxyl group of hydroxide layer and possible presence of interlayer water. The bands at 1003 cm^−1^ are for the deformation mode of Al–OH group (M-OH). Upon incorporation of EPO into LDHs, the intensity of hydroxyl stretching vibration bands increased. Furthermore, carbonyl stretching ( −C = O) vibration of carboxylic acid appeared as an intense band at 1760 cm^−1^. After embedding EPO, the characteristics Al–OH band shifted to 1005 cm^−1^, indicating incorporation of EPO to the interlayers. In case of the EPO-INHs, the spectra presented all characteristics bands related to the EPO-LDHs along with the SA characteristics bands (Fig. 3B).

The XRD patterns of LDHs and EPO-LDHs are shown in Fig. 3C. The characteristic peaks of LDHs corresponding to the atomic planes at (003) and (006) indicate ordered stacking of the layers. These intense reflections match those of LDHs synthesized using the coprecipitation method, as reported previously [68], implying the successful synthesis of LDHs. Notably, the intercalation of EPO into LDH layers caused a shift in the major diffraction peaks to lower angles, confirming the successful encapsulation of EPO within the interlayer spaces of LDHs. This result aligns with a previous report on LDHs encapsulated with anionic drugs like fluvastatin and pravastatin, where diffraction lines shifted to lower angles due to an increase in basal spacing [69]. Considering both the previous reports and the XRD results observed in this study, it is evident that EPO was loaded into the interlayer spaces of LDHs.

The thermal stability of conventional hydrogel (Alg-Gel) and its nanocomposite counterpart INHs-0.25 was determined by TGA. The TGA curves of Alg-Gel and INHs are presented in Fig. 3D. INHs showed slightly better thermal stability comparison to Alg-Gel, which resulted in 50% weight loss at 500 °C. The TGA curves indicate that the inclusion of LDHs reduces the weight loss, implying a strong interaction between LDHs and SA. Furthermore, this interaction effectively inhibits decomposition at the early stage. In general, the weight loss of the hydrogel biomaterials in the temperature range between 50 °C to 500 °C can be divided into three stages. In the first stage, the weight loss in the thermogravimetric curve was mainly due to the loss of water in the biomaterials. As the temperature raised, the biomaterials entered into the second stage weight loss. At this stage, the weight loss occurred by the decomposition of functional groups in the alginate. The presence of -COOH and -OH groups are decomposed at the temperature between 200 °C and 350 °C. At this point, the decomposition rate was high for Alg-Gel. Interestingly, INHs showed slightly better stability, which indicated the improved thermal stability of INHs. This can be explained by the ionic interaction between cationic Al^3+^ and Mg^2+^ ions and anionic carboxylic groups in the LDHs and alginate can possibly reduce the decomposition.

### Mechanical properties

**Fig. 4:**
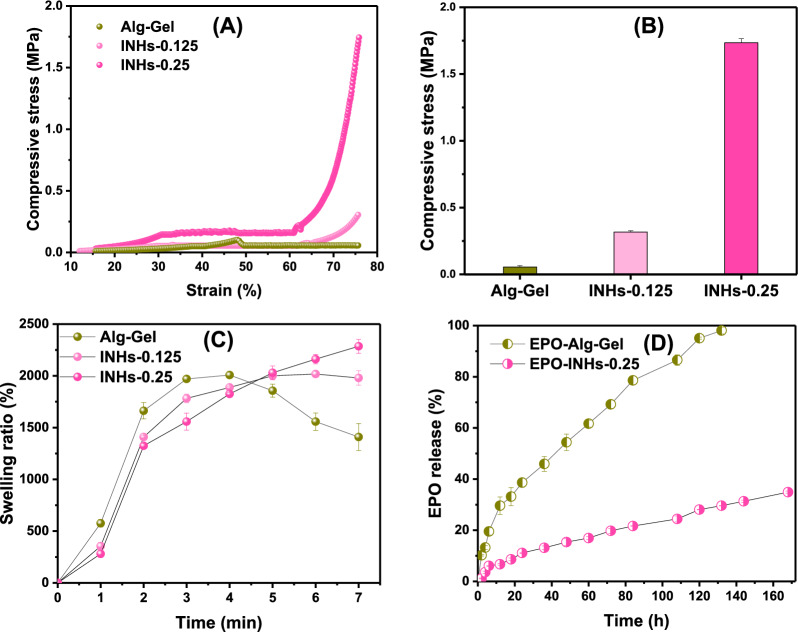
Mechanical properties of hydrogel. **A** The compressive stress–strain curve of hydrogels. **B** Compressive strength of different of hydrogels. **C** The swelling ratio of hydrogels as a function of time. **D** In vitro EPO release profile of hydrogel systems in PBS solution. Error bars in the graph represents mean ± SD (n = 3)

The compressive mechanical property of hydrogel is a crucial parameter because of the forces experienced with surrounding tissues [70, 71]. Thus, the unconfined compression test was conducted to investigate the effect of LDHs nanoparticles on the compressive strength of hydrogels. Figure 4A presents the compressive stress–strain (σ−ε) curves of INHs and Alg-Gel. It can be observed from the stress–strain curves that the INHs hydrogels with high LDHs exhibited a higher compressive strength (Fig. 4B). Regardless of the amount of LDHs, the mechanical strength of INHs was significantly higher than that of Alg-Gel. Precisely, the compressive stress of INHs-0.25 and INHs-0.125 was found to be 1.75 MPa and 0.31 MPa, respectively; whereas the compressive stress of Alg-Gel under the maximum strain of 75% was 0.056 MPa.

### Swelling behavior

The swelling property of hydrogels play an important role in the controlled drug delivery systems. Swelling of hydrogels depends on cross-linking density, porous structures as well as physical interactions within the porous networks. To evaluate the effect of LDHs on the swelling behavior of hydrogels, the gels were prepared with different LDHs concentrations and compared with Alg-Gel. After 120 s of incubation in PBS, the hydrogel formulation showed effective swelling and the LDHs in the hydrogels had little influence in swelling pattern (Additional file 1: Fig. S3 and Fig. 4C). Initially, the dried Alg-Gel group rapidly absorbed a great amount of water and the swelling ratio reached around 1700% within 2 min. This is due to the high number of polar groups in the network, including hydroxyl groups and carboxylate ions [72–74]. Further incubation of Alg-Gel led to the disassembly of the hydrogel network and the swelling ratio start decreased, which can be explained by the poor networking nature of Alg-Gel. At the same time, the gels with higher LDHs amount swelled less than gels with lower or no LDHs. In particular, the swelling ratio of INHs continue to increase and reached 2300% within 7 min and then attained plateau. The swelling ratio of INHs prepared in this study was higher than alginate hydrogels prepared with cellulose nanocrystals [75, 76].

### In vitro EPO release

In this experiment, LDH-free and LDH-reinforced Alg-Gel were used to encapsulate EPO and its controlled release pattern was examined in PBS medium as a function of time. It can be clearly observed from Fig. 4D that the EPO-INHs-0.25 system has a significantly slower release than that of the EPO-loaded in conventional Alg-Gel. As expected, Alg-Gel showed burst release of EPO and released 10% of proteins just after 2 h. Overtime, EPO release steadily increased and complete release was observed in 132 h. Remarkably, the burst release was significantly reduced with EPO-INHs and the EPO release was sustained. INHs released 30% of EPO after 132 h compared to complete EPO release with Alg-Gel. It should be noted that INHs exhibited more control in EPO release and released only 40% even after one week. This controlled release pattern indicates that incorporation of LDHs in the hydrogel can control the release protein therapeutics.

### 3D printing of patch (Lattice) structures with INHs

Improved mechanical property and controlled swelling behavior of INHs make it suitable to be deployed as 3D printable ink and applied in various tissue engineering applications [12]. In general, many alginate-based inks exhibit low viscosity and inadequate mechanical strength, resulting in filament collapse and instability in the 3D-printed constructs. Therefore, it is anticipated that the incorporation of INHs with exceptional mechanical properties could not only enhance the printability and post-printing structural stability of alginate inks but also various other aspects such as cell adhesion, porosity, and degradative properties. To assess the printability of Alg-Gel and INHs, samples were loaded onto a dispensing printhead that employed extrusion technology for printing. The extrusion process was conducted at room temperature, making this technology applicable to cell-incorporated bioinks as well.

**Fig. 5:**
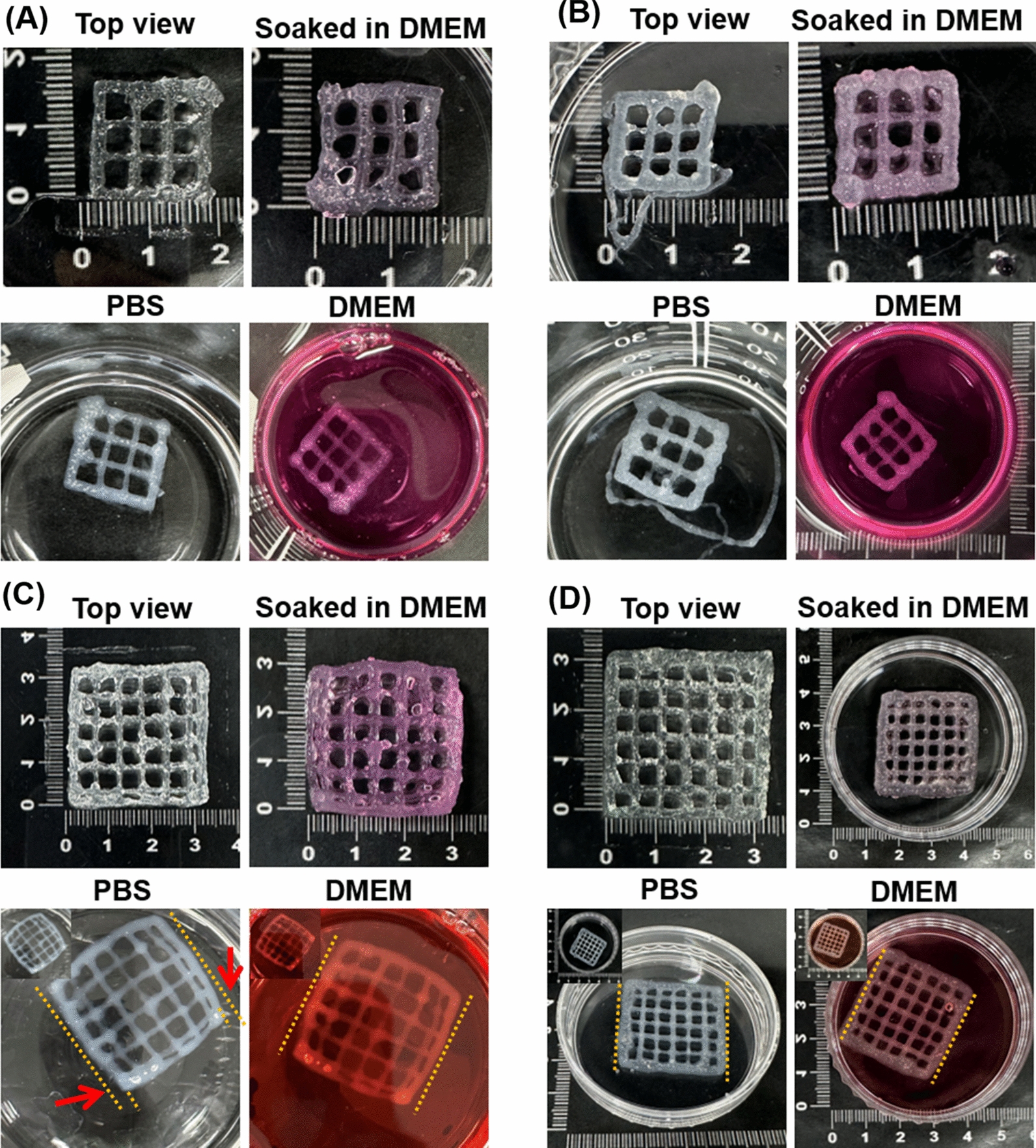
3D printing of the hydrogels. **A** and **C** Macroscopic images of 3D printed Alg-Gel grids of different sizes (15 mm × 15 mm and 30 mm × 30 mm). **B** and **D** Macroscopic images of 3D printed INHs-0.25 grids of different sizes (15 mm × 15 mm and 30 mm × 30 mm). The grids were printed in 6 wt.% Alg or INHs-0.25 with 0.1 wt.% CaCl_2_ concentrations and subsequently soaked in DMEM and PBS to examine the stability

As shown in Fig. 5, the printed patch (lattice) structures (15 mm × 15 mm) exhibited uniform filaments and the structural integrity was retained after incubation with water, PBS and cell culture medium. On increasing the scaffold dimensions to 30 mm × 30 mm, the integrity of the INHs-scaffolds was well-maintained while Alg-Gel filaments tend to disperse due to the inadequate networking within the hydrogels. A primary limitation of utilizing alginate solely for printing lies in the potential for uncontrolled swelling and slight shape alteration during post-printing procedures. This phenomenon (curved filament struts) is evident in Fig. 5C (PBS and DMEM). In contrast, the inclusion of INHs within the gels prevented any shape transformation when compared to the control samples. The provided guidelines serve to indicate the intended shape of the printed structures following incubation in both PBS and DMEM. Further, INHs gels provide added stability to the printed structure due to the presence of multiple ions and the intercalations.

### In vitro biocompatibility

**Fig. 6:**
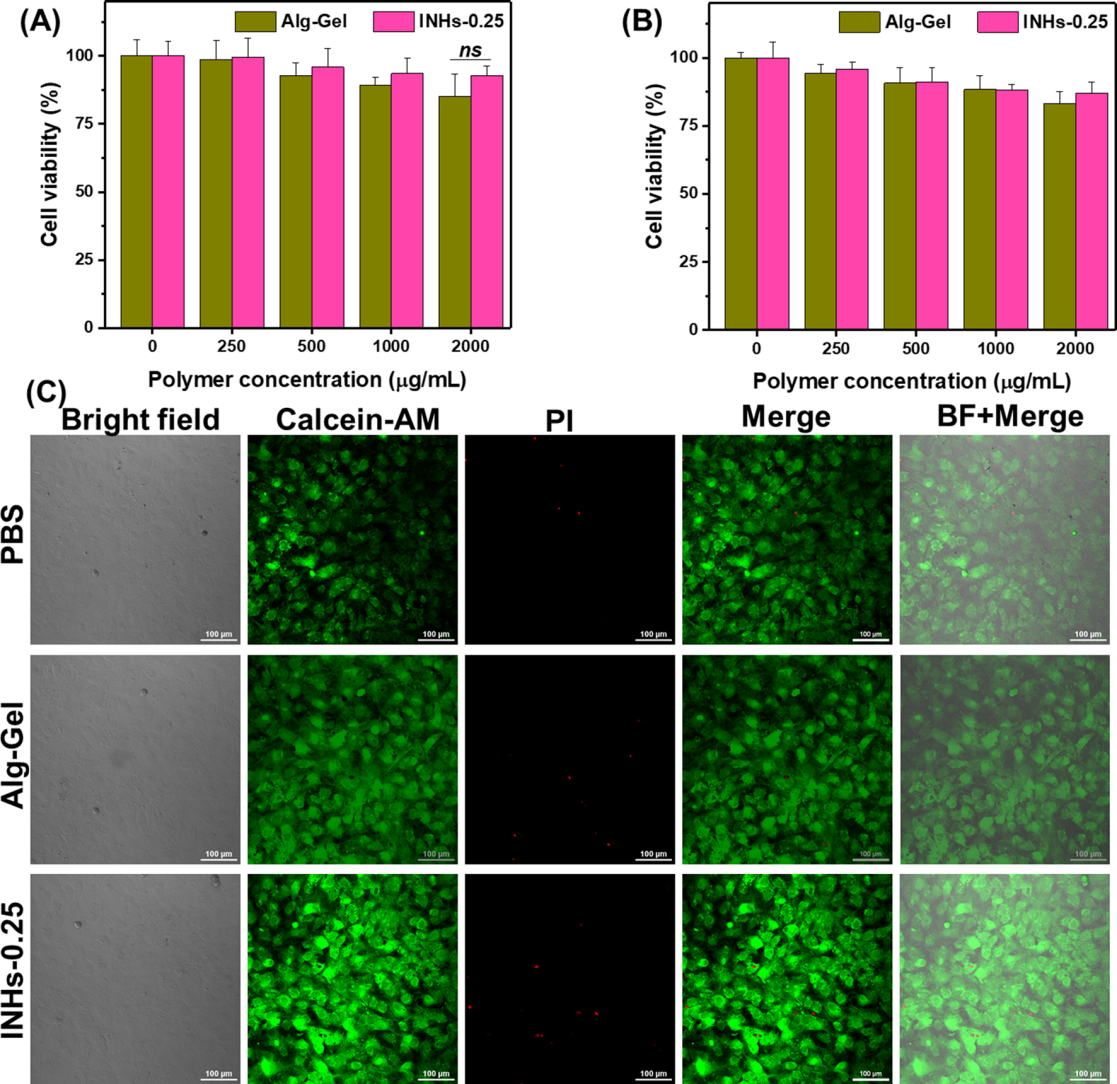
In vitro biocompatibility test. **A** and **B** Biocompatibility of HaCaT keratinocytes and BLO-11 mouse muscle fibroblast cells incubated with hydrogel formulation for 24 h. **C** Live and dead images of BLO-11 mouse muscle fibroblast cells stained by calcein-AM and propidium iodide (PI) (green (calcein-AM): live cells; red (PI): dead cells) observed using confocal laser scanning microscopy

Injectable hydrogels, which are administered in the body, have to be safe for their use. Therefore, they need to be tested carefully for toxicity and biocompatibility [77]. The in vitro biocompatibility of the various concentrations of Alg-Gel and INHs-0.25 were evaluated with MTT assay using HaCaT and BLO-11 cells. The cell viability of both HaCaT and BLO-11 cells remained over 80% even when exposed to 2000 μg of polymer (Fig. 6A, B). The slightly high proliferation of INHs can be explained that INHs could release nutrient substance that required for cell growth during hydrogel biodegradation, which led to the accelerated cell growth. In addition, the presence of ions in the LDHs can promote the cell proliferation [78]. It has been previously demonstrated that strontium ions promote human bone marrow stromal cell proliferation [79].

In order to further confirm the biocompatibility of hydrogels, confocal laser scanning microscopic images of cells were imaged after staining with LIVE/DEAD staining kit (Fig. 6C). For imaging, BLO-11 mouse muscle fibroblast cells were incubated with Alg-Gel and INHs-0.25 precursors for two days. Interestingly, some of the cells showed clear pseudopodium which indicates effective cell communication, induced cell spreading and attachment.

### In ovo vascularization assessment of INHs

**Fig. 7:**
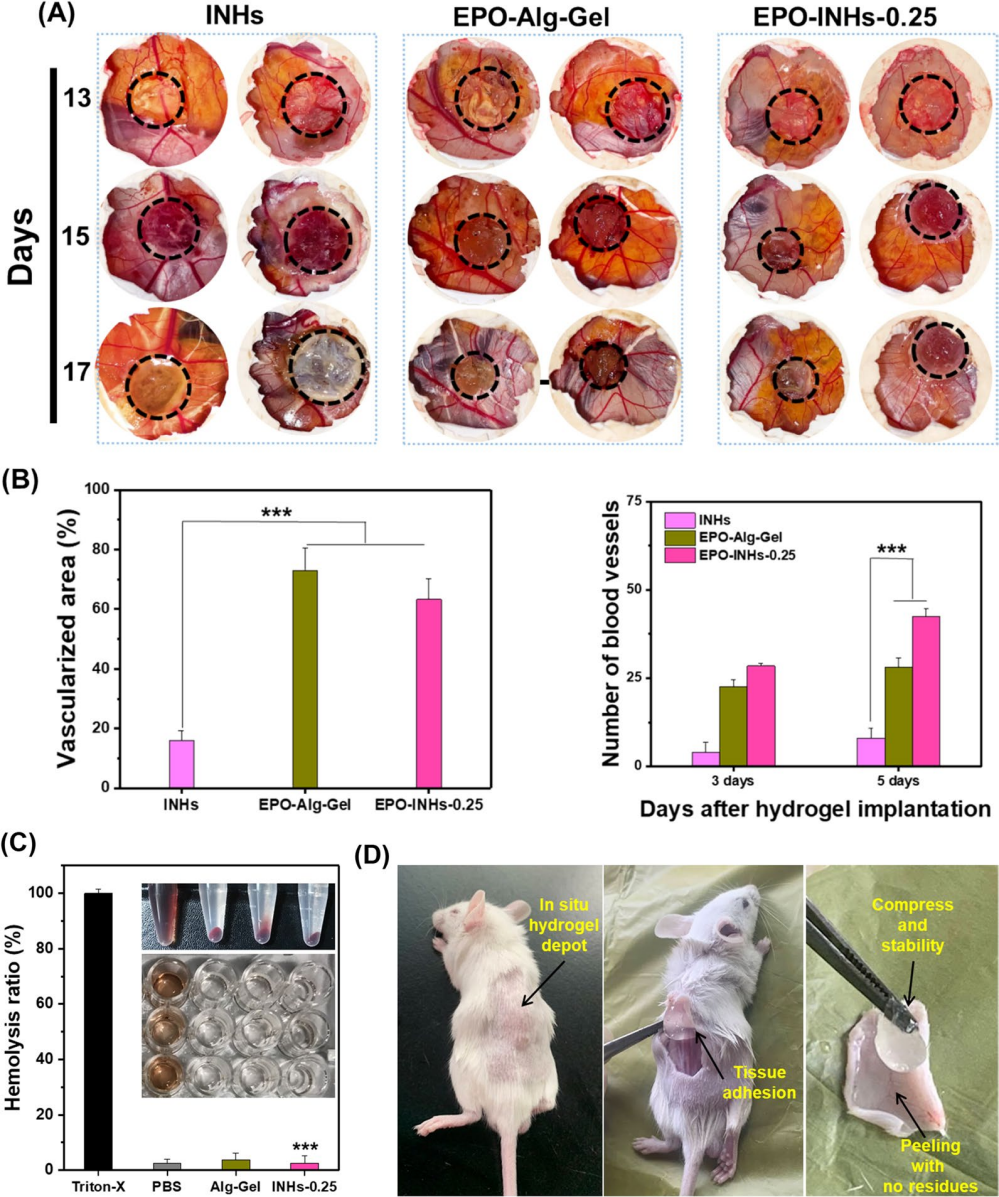
Angiogenic potential of EPO-loaded hydrogels. **A** Images of the hydrogels placed in the CAM at 13 days of chick embryo. The angiogenic potential of the hydrogel formulation was examined by imaging at 15 and 17 days. Sustained release of EPO from the hydrogels induced the angiogenesis in a CAM assay. **B** Quantification of vascularized area and blood vessels. **C** Blood compatibility of the hydrogels was examined using hemolysis test. Inserted pictures are for RBCs treated with different hydrogels. **D** In situ injection of INHs into the subcutaneous region of albino mice. A bump appeared under the mouse’s skin immediately after the injection. Stability assessment of in situ formed hydrogels was examined by sacrificing the mice. Error bars in the graph represents mean ± SD (n = 3). Asterisks (*) denotes statistically significant differences. ^*^P > 0.05; **P < 0.01; ***P < 0.001

This test is to aimed to confirm the angiogenic properties on a chick CAM through observation of chick embryo development (Fig. 7A). In general, chick embryos grew healthily with hydrogels placed on their CAMs. More importantly, on day 15 of being implanted, the egg group of EPO-Alg-Gel saw a great increase in vascularized area and the level of vascularization seemed to develop fully as they did not change much on day 17 at 73.03% (Fig. 7B). This result clearly revealed that EPO strongly promoted vascularization. EPO-INHs-0.25 also promoted vascularization but comparatively slow and gradual, with a vascularized area of 56.9% (in comparison to the INHs-0.25 group which showed a vascularized area of 16.04%). The number of blood vessels in the vascularized area was calculated to examine the effect of controlled EPO release. As shown in Fig. 7C, the number of blood vessels in the EPO-INHs-0.25 implanted formulation was 29 on Day 13 and increased to 43 on Day 15. On the other hand, there was no significant increase in the number of blood vessels for EPO-Alg-Gel group (23 and 28 on Day 13 and Day 15 respectively).

### Hemocompatibility of hydrogels

Blood contact is unavoidable for hydrogels used in biomedical applications. Therefore, it is important to investigate hemocompatibility of INHs before implanting into the animal model. Figure 7C shows the hemolysis ratio of RBCs exposed with Alg-Gel and INHs; inserted macroscopic images correspond to RBCs suspended in different hydrogels. PBS and Triton-X were used as negative and positive controls respectively. The supernatant of PBS, Alg-Gel and INHs are clear with pellets of RBCs at the bottom. In particular, RBCs found to be more compatible when exposed with INHs-0.25. As expected, Triton-X treated group showed bright red color supernatant with no RBC pellet at the bottom, indicating that RBCs were ruptured.

### In vivo hydrogel formation and biodegradation

A small bump appears on the back of the mouse after implantation as shown in Fig. 7D. It is clear that a stable gel was formed instantly under the skin without spreading to the neighboring site. The in vivo experiment demonstrated the successful utilization of INHs-0.25 as an injectable biomaterial with an optimal gelation time and appropriate gel formation on subcutaneous implantation.

**Fig. 8:**
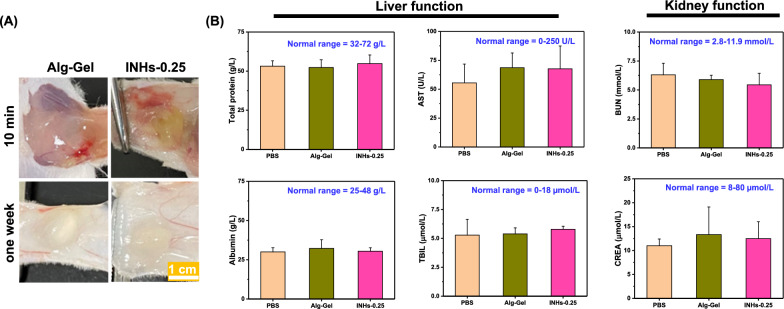
In vivo safety of INHs. **A** In vivo biodegradation of INHs and Alg-Gel. To examine biodegradation, hydrogels (100 µL, 4 wt.%) were injected into the back of mice and recovered after one week. **B** Blood biochemistry analysis of mice implanted with hydrogels one week after injection. AST: aspartate transaminase; *TBIL* Total bilirubin, *BUN* Blood urea nitrogen; and *CREA* creatinine

To verify the biodegradation, we subcutaneously implanted the hydrogel at the back of mouse and cut open after one-week to assess the biodegradability of INHs (Fig. 8A). Notably, the control Alg-Gel lacking LDHs exhibited rapid biodegradation. Interestingly, INHs displayed sustained biodegradation, with a slightly slower degradation rate compared to Alg-Gel. From the quantitative analysis, it is clear that the biodegradation of INHs was slower than that of Alg-Gel (Additional file 1: Fig. S4). This phenomenon primarily arises from the presence of LDHs within the hydrogels, which enhances their stability and renders them suitable for various biomedical applications, including hard tissue engineering. In addition, blood samples also collected for the biochemical analysis. Biochemical analysis demonstrated that there is no significant difference in the blood biochemical parameters of INHs and Alg-Gel injected mice when compared with control mice group (Fig. 8B). This result concludes that there is no obvious organ damage upon implantation of INHs.

## Discussions

Although a number of 2D nanomaterials have been developed to improve mechanical properties of hydrogels, the utilization of lamellar inorganic solids with a brucite-like structure for controlling the release of protein therapeutics has not been investigated in detail. In this work, a nanoengineered injectable system formulated by incorporating 2D LDH clay materials with a large surface area into alginate hydrogels was developed for controlled release of therapeutic proteins. The nanoengineered injectable hydrogels (INHs) demonstrated quick gelation, injectability, and excellent adhesion properties on human skin. Also, in morphological analysis, the gels demonstrated good porosity. The combination of these ionic and hydrophobic interactions strengthens the molecular entanglement between SA and LDHs, resulting in the formation of a robust physically cross-linked hydrogel network. This network exhibits strong mechanical properties and can retain its structural integrity even in the absence of chemical crosslinking agents.

During the mechanical analysis, the dramatic increase in compressive properties of INHs-0.25 was attributed to ionic interaction between negatively charged SA polysaccharides and positively charged layer-structured LDHs. Further, enhancement of hydrogen bonding, together with intercalation of polymers into galleries of LDHs contributed to the enhanced mechanical strength of INHs [80, 81]. When the hydrogel was compressed, the polymer network was densified and compacted, triggering a considerable increase in intermolecular interaction [82]. Therefore, the ultimate compressive strength of hydrogels can be controlled by adding LDHs to the Alg-Gel matrix. It should be noted that INHs-0.5 showed relatively poor dispersion and extrudability and hence INHs-0.5 excluded was from further experiments. Short gelation time, high compressive strength, and controlled swelling and release properties of INHs-0.25 made it the suitable candidate for further experiments including 3D printing, *in ovo*, and in vivo studies.

Swelling studies: Generally, the structure of INHs is not only formed by physical cross-linking between Ca^2+^ and –COO^−^ groups but also reinforced by electrostatic interaction between positively charged metal ions of LDHs and SA. Thus, the presence of LDHs probably forms a tight network that limits the expansion of pores leading to decreased water uptake. However, when the pure Alg-Gel swell over their capacity, they start to break out and release water molecules, which results in decreased swelling ratio after 4 min. Further incubation of Alg-Gel in PBS continues to show decrease in swelling ratio, which indicated that hydrogel network broken at the early stage cannot reassemble due to the poor networking. Interestingly, INHs were stable and INHs with higher LDHs content still maintain water in their structure during this period of time, indicating that incorporation of LDHs inside the gel matrix control the water uptake in the hydrogels.

In in vitro release studies, the burst release was significantly reduced with EPO-INHs and the EPO release was sustained. INHs released 22% of EPO after 108 h compared to 85% EPO release with Alg-Gel. This controlled release pattern indicates that incorporation of LDHs in the hydrogel can control the release protein therapeutics.

The INHs formulations were successfully 3D printed with high resolution, and the resulting scaffolds retained their initial structure without significant deformation or distortion. The shape fidelity of the printed structures was intact even after incubation in cell culture medium. This demonstrates the possibility of these INHs to be applied as a patch for delivery of different drugs similar to the 3D printed patches have been reported recently [83, 84].

In MTT assay, cell growth seems to be higher in INH structure than that of the Alg-Gel. This can be easily explained by the fact that INH provides a larger surface for the cells where LDH nanoparticles are dispersed on the internal and external surface of the highly porous hydrogel.

In case of Live/Dead assay, the extent of cell growth and spreading in the INHs-0.25 was high in comparison to cells treated with only Alg-Gel and control. The presence of LDHs in the hydrogel formulation seems to be beneficial to cells in terms of larger surface area for cell adhesion.

In ovo vascularization assessment results confirm that with the presence of LDHs, there was more control of EPO being released from the hydrogel and EPO is a main factor contributing to significant growth of blood vessels. Controlled release of EPO from INHs facilitated superior angiogenic potential *in ovo* (chick chorioallantoic membrane) compared to Alg-Gel. The results are in line with other reports that highlight the potential pro-angiogenesis properties of LDHs [85]. Being pro-angiogenic, INHs can be explored as promising implantable scaffolds in mammalian models for their regenerative potential. From the hemocompatibility test as well as biocompatibility test, it is clear that INHs are hemo- and biocompatible and can be used as injectable hydrogels that could not only control the release of bioactive agents but also modulate the microenvironment of various defects. When subcutaneously implanted in albino mice, the INHs formed a stable gel in vivo without inducing any adverse effects. The results suggest that the proposed INHs in this study can be utilized as a minimally invasive injectable platform or as 3D printed patches for the delivery of protein therapeutics to facilitate tissue regeneration.

## Conclusions

In summary, nanoengineered injectable hydrogels (INHs) were developed by combining two-dimensional layered double hydroxide (LDH) clay materials and sodium alginate (SA) for controlled release of a model glycoprotein hormone, erythropoietin (EPO). The INHs showed rapid and tunable gelation properties because of the incorporation of charged and disc-shaped layers of LDHs that could electrostatically bind and interconnect the network, subsequently allowing rapid formation of gels. The mechanical property of hydrogels was tuned by the presence of LDHs, which is not only important in controlled delivery applications but also play a prominent role in tissue engineering applications. The EPO embedded within the interlayer spaces of LDHs effectively controlled its release from the INHs, leading to a prolonged duration of release. The INHs had good biocompatibility to various cells tested in this study. The excellent biocompatibility and controlled release of EPO exhibited superior angiogenic potential in *in ovo* experiments. Moreover, the INHs demonstrated the ability to form a stable gel in vivo. In vivo biodegradation and biochemical analysis demonstrated that INHs are safer and suitable for implantable biomaterials. These findings suggest that the INHs hold great promise as a viable option to address the practical requirement for customizable drug delivery depot in various tissue engineering and regenerative medicine applications.

### Supplementary Information

**Additional file 1: Fig. S1.** Pore diameter and pore size distribution of Alg-Gel and INHs. The data were collected from SEM images using ImageJ software. **Fig. S2.** SEM image of INHs with sonication and the corresponding elemental mapping analysis of Al and Mg. **Fig. S3.** Swelling behavior of hydrogels. **Fig. S4.** Biodegradation of pattern of hydrogel was estimated after freeze drying the recovered hydrogels in Fig. 8A. The extent of biodegradation was measured using the mass loss method.

## Data Availability

The data presented in this study are available on request from the corresponding author.

## Abbreviations

3D: Three-dimensional
SA: Sodium alginate
LDH: Layered double hydroxide
INH: Nanoengineered injectable hydrogels
EPO: Erythropoietin
SD: Sprague–Dawley
RBCs: Red blood cells
CKD: Chronic kidney disease
rhEPO: Recombinant human erythropoietin
SEM: Scanning electron microscopy
CAM: Chick embryo chorioallantoic membrane (CAM) assay
MTT: (3-(4, 5-Dimethylthiazolyl-2)-2, 5-diphenyltetrazolium bromide)
MgCl_2_.6H_2_O: Magnesium chloride hexahydrate
AlCl_3_.6H_2_O: Aluminum chloride hexahydrate
CaCl_2_: Calcium chloride
FTIR: Fourier transform infrared
TEM: Transmission electron microscopy
XRD: X-ray diffraction
TGA: Thermogravimetric analysis
DMEM: Dulbecco's modified eagle medium
PBS: Phosphate buffered saline
