# Supplementary material for: Nanoengineered injectable hydrogels derived from layered double hydroxides and alginate for sustained release of protein therapeutics in tissue engineering applications
Source: J Nanobiotechnology. 2023 Nov 2;21:405. doi: 10.1186/s12951-023-02160-2 (PMC10623704; doi:10.1186/s12951-023-02160-2)
Supplement: Supplementary file 1 — Additional file 1: Fig. S1. Pore diameter and pore size distribution of Alg-Gel and INHs. The data were collected from SEM images using ImageJ software. Fig. S2. SEM image of INHs with sonication and the corresponding elemental mapping analysis of Al and Mg. Fig. S3. Swelling behavior of hydrogels. Fig. S4. Biodegradation of pattern of hydrogel was estimated after freeze drying the recovered hydrogels in Fig. 8A. The extent of biodegradation was measured using the mass loss method. [file 12951_2023_2160_MOESM1_ESM.docx]

**Additional file Information**

**Nanoengineered injectable hydrogels derived from layered double hydroxides and alginate for sustained release of protein therapeutics in tissue engineering applications**

V.H. Giang Phan ^a^, Hai-Sang Duong ^a^, Quynh-Giao Thi Le ^a^, Gopinathan Janarthanan^b^, Sanjairaj Vijayavenkataraman^b,c^, Hoang-Nam Huynh Nguyen ^a^, Bich-Phuong Thi Nguyen ^a^, Panchanathan Manivasagan^d^, Eue-Soon Jang^d^, Yi Li^e^*, and Thavasyappan Thambi ^f^*

^a^ Biomaterials and Nanotechnology Research Group, Faculty of Applied Sciences,

Ton Duc Thang University, Ho Chi Minh City, Vietnam

^b^The Vijay Lab, Division of Engineering,

New York University Abu Dhabi, Abu Dhabi, United Arab Emirates

^c^Department of Mechanical & Aerospace Engineering, Tandon School of Engineering,

New York University, Brooklyn, NY 11201, USA

^d^ Department of Applied Chemistry, Kumoh National Institute of Technology,

Daehak-ro 61, Gumi, Gyeongbuk, 39177, Republic of Korea

^e^ College of Materials and Textile Engineering & Nanotechnology Research Institute,

Jiaxing University, Jiaxing 314001, Zhejiang Province, PR China

^f^ Graduate School of Biotechnology, College of Life Sciences, Kyung Hee University,

Yongin si, Gyeonggi do 17104, Republic of Korea

*Corresponding authors:

Thavasyappan Thambi, Ph.D.

Tel.: +82-31-290-7393; Fax: +82-31-299-6857; e-mail: [thambi@khu.ac.kr](mailto:thambi@khu.ac.kr)

Yi Li, Ph.D.

Tel.: +86-0573-8391-3364; Fax: +86-0573-8364-2282; e-mail: [liyi@zjxu.edu.cn](mailto:liyi@zjxu.edu.cn)

**Procedure to quantify vascularized areas and blood vessels:**

The steps involved in quantifying vascularized areas and blood vessels by using ImageJ as follows:

**Step 1:** Open ImageJ software → Import an image: File – Open – Choose the picture.

**Step 2:** Set the scale for the imported picture: Draw a line based on a scale bar on the picture → Choose the functions “Analyze” – “Set scale” → In the box “Set scale”, adjust 2 parameters: “Known distance” and “unit of length” according to the scale bar of the picture.

**Step 3:** Choose “Image” – “Type: 16 Bit” to convert the color picture into a suitable white-black image.

**Step 4:** Choose “Process” – “Subtract Background” to remove the unnecessary areas and keep branches of blood vessels.

**Step 5:** Choose “Image” – “Adjust” – “Threshold...” and then adjust to select the red area based on the blood vessels.

**Step 6:** Go to “Process” – “Binary” – “Make Binary” and then choose “Process” – “Noise” – “Remove outliners” to remove the unrelated points.

**Step 7:**

- **To measure vascularized area:** Choose “Analyze” – “Measure” to measure the vascularized areas. If the size of the blood vessels increases, the measured vascularized area increases. Therefore, using ImageJ software to calculate the percentage of the increased vascularized area.
- **To measure numbers of blood vessels:** Choose “Process” – “Binary” – “Skeletonize” and then choose “Analyze” – “Skeleton” – “Analyze Skeleton” → The result: in the first row, it shows a total of branches.

*Note: the symbol “...” show toolbars in ImageJ software.


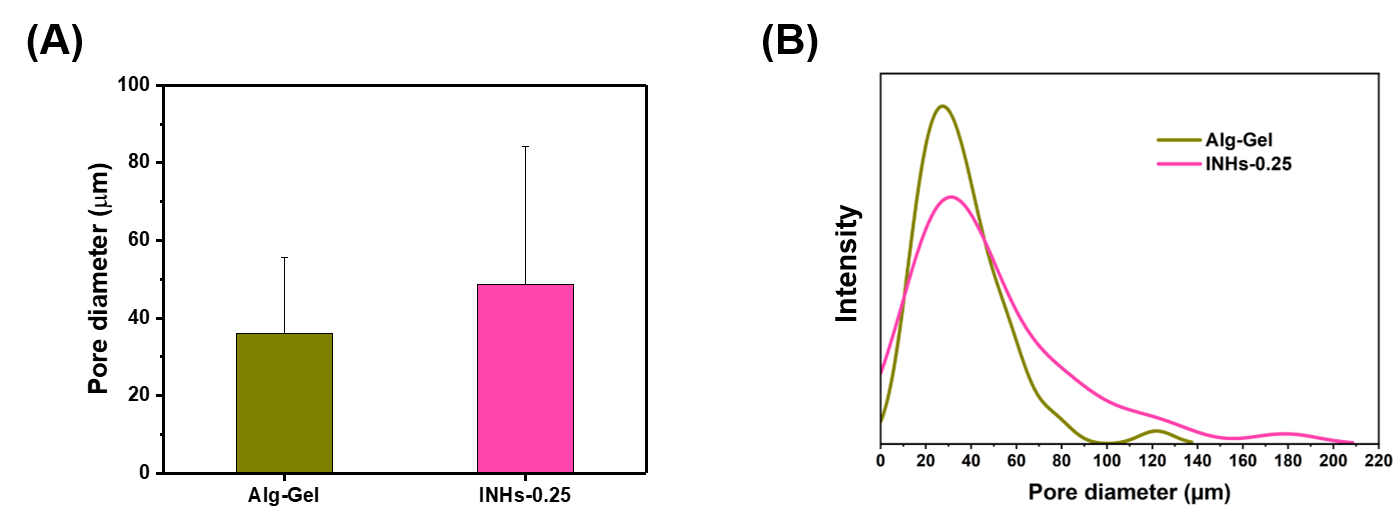


**Fig. S1.** Pore diameter and pore size distribution of Alg-Gel and INHs. The data were collected from SEM images using ImageJ software.


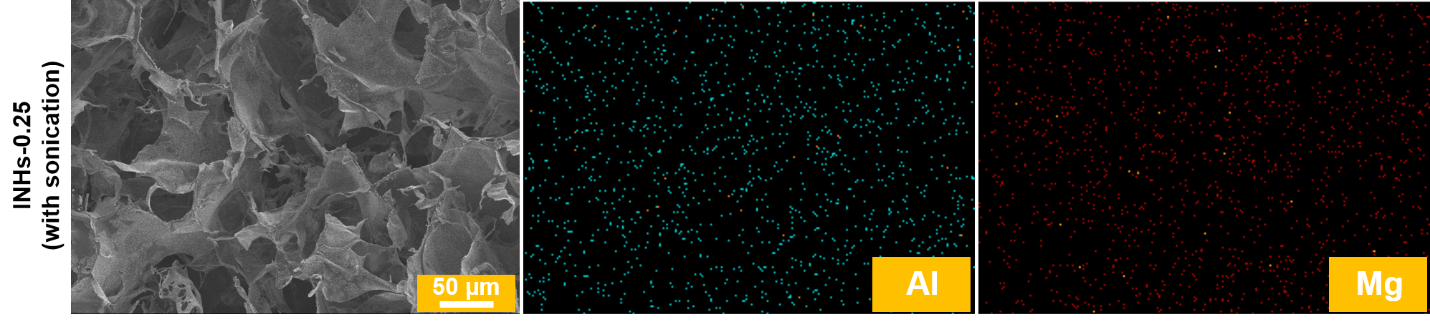


**Fig. S2.** SEM image of INHs with sonication and the corresponding elemental mapping analysis of Al and Mg.


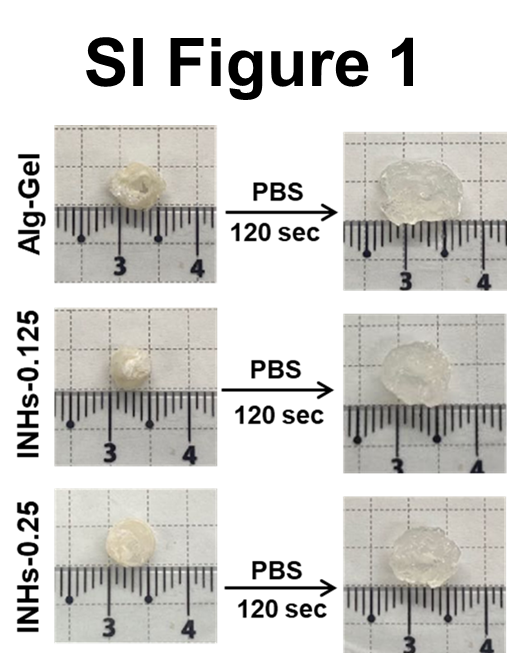


**Fig. S3.** Swelling behavior of hydrogels.


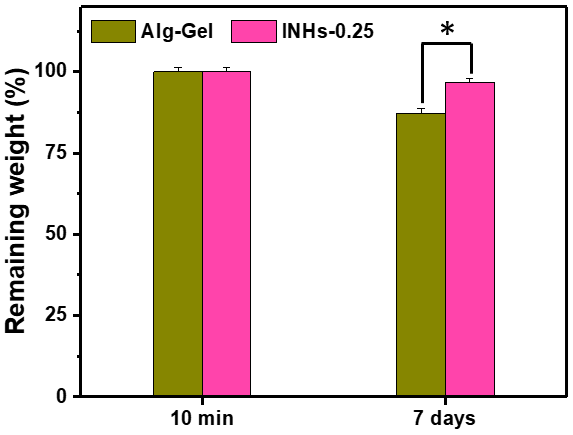


**Fig. S4.** Biodegradation of pattern of hydrogel was estimated after freeze drying the recovered hydrogels in Fig. 8A. The extent of biodegradation was measured using the mass loss method.
